# Amputation and Extirpation of the Uterus; with Remarks on This Operation, and Its Results

**Published:** 1829-01-01

**Authors:** 


### XXXVII.

### Amputation and Extirpation of the Uterus.

In various Numbers of this Journal we have given accounts of the above very formidable operation, which has not, till of late years, been performed in this country. We fear that British experience has now decided against the repetition of the operation, by any man who has reputation to lose; or, who conscientiously weighs the uncertainty of diagnosis, and the sufferings of the patient against the prospect of success. The operation has now been performed five times in this country — thrice by Dr. Blundell—once by Mr. Banner—and once by Mr. Lizars. Of the operations performed by Dr. Blundell, one only has been published—and that the successful one. It is stated in the Medical Gazette, that in the first case, the patient died—and if so, we call upon Dr. Blundell, in the name of humanity and science, to publish that case—and we hope Dr. Blundell will also give satisfactory reasons why the unsuccessful case was not published before this time? We have heard many of our brethren inquire, whether Dr. Blundell has preserved (but how can this be doubted?) the uterus which was removed in the case where the patient survived; and whether he will favour the inquisitive pathologist with a sight of the preparation? It is of great importance to know, whether, in these successful cases, the uterus was *really* cancerous—and we cannot, for a moment, doubt that Dr. Blundell will be happy to exhibit the diseased organ to any of his professional brethren who may be curious to examine it. In the third case, operated on by Dr. Blundell, we understand the patient expired in nine hours after the operation.

In Mr. Banner's case, the operation was performed on the second of September, in the presence of several medical gentlemen, at Liverpool, and appears to have been dexterously managed; nevertheless the patient died on the fourth day. Mr. B. states, that the uterus was much larger than natural, and that "several *tubercles* of various sizes were loosely attached to the body and fundus." When we consider how very rarely carcinoma and tubercles are found in the same organ, we cannot but doubt the existence of real cancer of the womb, in the foregoing case, though "ulceration had taken place on the os uteri," and "a section of the uterus exhibited the common appearances of a scirrhus."

The operation performed by Mr. Lizars is not yet decided; (20th Oct.) but we much fear that, if the whole uterus has been removed, the event will be fatal. It is now three years since we freely expressed our opinion on this operation. In the third Volume of the present Series, p. 264, the following passage occurs. "*We consider the extirpation of a uterus, not previously protruded or inverted, one of the most cruel and unfeasible operations that ever was projected or executed by the head or hand of man. We are very far from discouraging bold or untried operations; but, there is a line, beyond which it may not be prudent to go, even should a solitary instance or two of success rise up as precedents to bear out the operator.*" *July*, 1825. Sincerely do we regret that our prognostications have been but too truly confirmed by the attempts of bold, though perhaps incautious practitioners of the present day. We cannot believe that the total ablation of the uterus will ever again be attempted in this country.

P. S. Since the above remarks were written, we have received Mr. Ashwell's new Work on Parturition, in which Dr. Blundell has given a summary account of the first and third, or unsuccessful operations. We here insert them.

"*Case* 1.—Mrs. A. B. æt. 33, the mother of six children, the last born *seven* years ago, of constitution naturally healthy, came under my observation, reduced by malignant disorganization of the neck and mouth of the uterus and upper part of the vagina. There was ulceration, flooding, copious watery and offensive discharge, the constitution was giving way, and it seemed probable life would not be protracted beyond one or two months. Assisted by Mr. Callaway and Mr. Martin of Horsham, I extirpated the uterus, together with the diseased portion of the vagina, the woman living for thirty-nine hours afterwards, but never thoroughly rallying. She expressed herself highly gratified with the relief of her central pains, but the skin remained clammy, the pulse ranged between 135 and 145 in the minute, small and weak, and there was a continual feeling of debility, mixed with that kind of composure which is so often observed at the fatal close of puerperal fever. Though no ligatures were applied, only six or eight ounces of blood were lost during the operation. The womb was as large as a goose's egg. All parties were candidly informed of the great danger of the operation before it was undertaken, and the patient herself was anxious that it should be attempted, as she felt without other hope. From examination after death it appears that the diseased mass was entirely removed, without any injury to the intestines, bladder, ureter, or urethra. Mr. Green and Mr. Callaway very carefully inspected the body. The bladder was fallen into the chasm, formed by the removal of the uterus, so that it lay upon the front of the rectum, and closed the head of the vagina. In the cavity of the pelvis there were two or three ounces of bloody serum, which might have been easily discharged by passing the finger between the bladder and rectum: the formation of adhesions was begun."

"*Case.* 3.—Mrs. ———, æt. 40, of dark complexion, spare make, and the mother of several children, was labouring under scirrhosity and thickening of the neck of the uterus and about a quarter of the vagina above, with some ulceration, and feeling herself in a state of rapid decay, she was, together with her friends, after the failure of other means, anxious that the operation should be tried.

"The vagina was lax and the uterus moveable. The dangers and the uncertainties inseparable from the removal of the uterus, in the present state of abdominal surgery, were candidly laid before all parties concerned. Mr. Green of St Thomas's Hospital, and Mr. Morgan of Guy's Hospital, considering that the constitution was not unfavourable for an operation of this kind, the patient still persevering in her wish, the parts consisting of the whole womb and the upper part of the vagina, were removed. When the sides of the vagina and broad ligaments were cut through, the principal hæmorrhage occurred, amounting perhaps to nine or ten ounces of venous blood. When the uterus was drawn down, the principal pain and collapse were produced. After the operation, the pulse became for a few minutes imperceptible at the wrist, afterwards gradually returning and ranging between 125 and 130 in the minute, with occasional though not frequent intermissions. Large doses of the tinct. opii were given, and the patient lay for the most part composed, with occasional slumbers: now and then tendency to restlessness was observed, although a complete rally could not be obtained. From the time of the removal of the parts the patient went on sinking, and died at the end of about nine hours, without scarcely a struggle. An examination instituted next day by Mr. Green and Mr. Morgan, proved, that the intestines, bladder, and ureters, remained uninjured. Some two or three ounces of clotted blood were found in the cavity of the pelvis, in a situation admitting of easy removal through the outlet. The womb was twice as large as in Mrs. Moulden's case, and the vessels, as appeared from examination of the womb itself and of the parts within the pelvis, from which it had been separated, were of considerable size, especially the veins. Death here seemed to be produced partly by the loss of blood, but mainly by the shock of the operation."*

* A fourth case has been recently alluded to by Dr. Blundell—also fatal.—*Ed.*

